# Dynamic thoracohumeral kinematics are dependent upon the etiology of the shoulder injury

**DOI:** 10.1371/journal.pone.0183954

**Published:** 2017-08-25

**Authors:** Juan López-Pascual, Álvaro Page, Pilar Serra-Añó

**Affiliations:** 1 Instituto de Biomecánica de Valencia, Universitat Politècnica de València, Valencia, Spain; 2 Departament de Física Aplicada, Universitat Politècnica de València, València, Spain; 3 Grupo de Tecnología Sanitaria del IBV, CIBER de Bioingeniería, Biomateriales y Nanomedicina (CIBER-BBN), València, Spain; 4 Departament de Fisioteràpia, Universitat de València, València, Spain; Universite de Nantes, FRANCE

## Abstract

Obtaining kinematic patterns that depend on the shoulder injury may be important when planning rehabilitation. The main goal of this study is to explore whether the kinematic patterns of continuous and repetitive shoulder elevation motions are different according to the type of shoulder injury in question, specifically tendinopathy or rotator cuff tear, and to analyze the influence of the load handled during its assessment. For this purpose, 19 individuals with tendinopathy and 9 with rotator cuff tear performed a repetitive scaption movement that was assessed with stereophotogrammetry. Furthermore, static range of motion (ROM) and isometric strength were evaluated with a goniometer and a dynamometer, respectively. Dynamic measurements of maximum elevation (Emax), variablility of the maximum angle (VMA), maximum angular velocity (Velmax), and time to maximum velocity (tmaxvel) were found to be significantly different between the tendinopathy group (TG) and the rotator cuff tear group (RTCG). No differences were found in the ROM assessed with goniometry and the isometric strength. The effect of increasing the load placed in the hand during the scaption movement led to significant differences in Emax, VMA, tmaxvel and repeatability. Therefore, only the dynamic variables showed sufficient capability of detecting differences in functional performance associated with structural shoulder injury. The differences observed in the kinematic variables between patients with tendinopathy and rotator cuff tear seem to be related to alterations in thoracohumeral rhythm and neuromuscular control. Kinematic analysis may contribute to a better understanding of the functional impact of shoulder injuries, which would help in the assessment and treatment of shoulder pain.

## Introduction

The prevalence of shoulder pain is estimated to be 15.4% in men and 24.9% in women [1]. This high prevalence has led to the use of special tools for assessing structural injuries of the shoulder, such as magnetic resonance imaging, radiography or ultrasound, in order to identify the etiology of the shoulder pain. Nevertheless, the treatment and rehabilitation of patients with shoulder pain involves not only knowing the diagnosis, but also assessing the impact of the injury on joint function. In effect, alterations in shoulder function have a critical impact on the performance of basic daily activities [2,3]. Moreover, there is no consistent correlation between the injury itself and the patient’s functional status [4,5], hence the importance of having techniques to assess the evolution of a patient’s functional status in a clinical context [3,4].

In clinical practice, the most commonly used techniques for assessing functional status are clinical scales and simple devices for measuring static variables, such as ROM or isometric strength assessment [6,7]. Despite their undoubted usefulness, these techniques do not provide information about the quality and accuracy of the movement. In contrast, continuous recording of 3D motion while performing functional gestures provides much more complete information about the ROM and the speed, smoothness and coordination of the movement. In the research context, video-photogrammetry is perhaps the most widely used technique for 3D motion capture and is considered the gold standard, as it is a non-invasive technique that provides very accurate and reliable measurements of kinematic variables [8–10].

Most clinical studies of 3D shoulder kinematics have focused on identifying differences between healthy individuals and those with shoulder injuries [11–15]. Studies aimed at quantifying differences in functional status associated with different conditions are scarcer and usually use only static measures, with few exceptions [16,17]. Moreover, most studies focus on scapular kinematics and scapulohumeral rhythm, since injuries directly affect these movements [18,19]. Although knowledge about scapular and glenohumeral kinematics is essential to understand the relationships between structural alterations and functional shoulder response, implementation of this in the clinical context has some disadvantages. Indeed, precise measurement of scapular kinematics in a clinical setting is difficult and is limited to 120° of humeral elevation due to the low reliability of the existing non-invasive systems when tracking scapular motion at larger angles of elevation [20,21]. Additionally, it has been observed that the different methods of analyzing scapular motion provide different results, which can lead to conflicting conclusions [17]. In contrast, the thoracohumeral movement is easy to measure reliably [10]. Since the kinematics of the humerus depend on scapulothoracic and glenohumeral movements [22], they could be affected by alterations in these elements of the kinematic chain. Therefore, thoracohumeral kinematics could be a useful indicator of the overall functional status of the shoulder. In this regard, there are published studies that use thoracohumeral rhythm to evaluate shoulder function in relation to activities of daily living (ADLs) [23,24]. Although these previous studies analyzed differences between the thoracohumeral kinematics of healthy people and individuals with an injury, so far no previous studies have analyzed differences in shoulder movement quality (characterized by variables such as velocity or movement variability) depending on the injury in question.

Obtaining kinematic patterns depending on the injury could be the key to achieving a more precise approach to the patient’s rehabilitation because it could help clinicians decide which interventions would be most appropriate. In this regard, it should be noted that the implementation of ADLs in clinical assessments is technically complex, due to the difficulty of establishing standardized procedures and the high variability in the execution of tasks, even in the case of the healthy population [25,26]. An alternative may be the measurement of simpler functional movements. In particular, shoulder scaption has been shown to be reliable [27] and effective in the assessment of functional alterations in patients with shoulder pain [13,16,21,28]. Furthermore, although it is a single motion, it is considered an important movement because it forms part of functional movement patterns [29].

The goal of this study is to explore whether the kinematic pattern within a thoracohumeral scaption movement, such as ROM, velocity, movement rhythm and variability differ if a shoulder has either tendinopathy or rotator cuff tear. We also analyzed the influence of the load handled during assessment of the kinematic pattern due to its underlying clinical interest and because it is more representative of the performance of ADLs [13,21]. Furthermore, we also investigated the effect of the injury on the variables of ROM, and isometric strength.

**Hypothesis:** variables derived from kinematic analysis of the thoracohumeral movement will be associated with poor movement kinematics and greater variability in people with rotator cuff tear than in people with tendinopathy. The information obtained from the other static variables, isometric strength and range of motion, will be unable to establish differences between these two injuries.

## Material and methods

### Participants

Patients were selected from a convenience sample and recruited over a six-month period. Participants were allocated to two groups. The tendinopathy group (TG) was composed of 19 patients with a chronic (> 3 months) rotator cuff tendinopathy without a full-thickness tear with a mean (SD) age of 46.89 (10.69) years. The rotator cuff tear group (RCTG) was composed of 9 individuals with chronic (> 3 months) full-thickness rotator cuff tears greater than 1cm^2^ in size, with a mean (SD) age of 57 (7.07) years. The size of the tears was between 1 cm^2^ and 3 cm^2^, which is classed as small to medium [30]. All the participants were right-handed and the injury was on their dominant side. The diagnosis was based on their magnetic resonance imaging and/or ultrasound scan results and was confirmed by their own physician. Exclusion criteria included (1) a history of previous surgery on the assessed shoulder; (2) previous fracture of the clavicle, scapula or humerus; (3) reproduction of symptoms during a cervical spine screening; and (4) known joint disease (e.g. rheumatoid arthritis or osteoarthritis).

**Fig 1:**
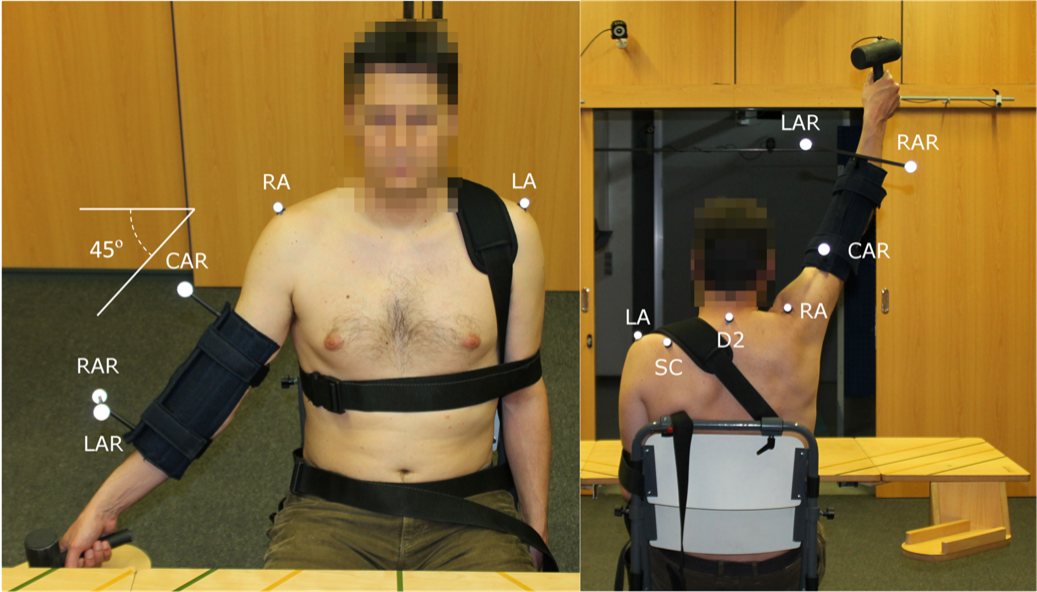
Marker and fastening setup. The image on the left is a front view of the marker setup and trunk fastening. On the right, a rear view of the marker setup and trunk fastening is shown. LA: Left acromion; RA: Right acromion; SC: Medial third of the scapular spine; D2: Second dorsal vertebra; CAR: Central arm; LAR: Left arm; RAR: Right arm.

All participants provided written informed consent, all procedures were conducted in accordance with the principles of the World Medical Association’s Declaration of Helsinki, and the protocols were approved by the Experimental Research Ethics Committee of Universitat Politècnica de València. Furthermore, the individual who appears in Fig 1 has given written informed consent (as outlined in the PLOS consent form) to publish these case details.

### Procedures

This is a cross-sectional study, and the procedures were performed in a single session by a blinded examiner in our laboratory. At each session, pain intensity, ROM, isometric strength and kinematic pattern of the shoulder were assessed in a random order using photogrammetry. The participants were allowed to rest for 5 minutes between assessments. Furthermore, the body mass index (BMI) was calculated taking into consideration the weight and height of each participants (kg·m^-2^). All the participants performed all the assessments.

### Pain intensity assessment

The pain intensity was evaluated at the beginning of the measurement session by a 100 mm visual analog scale (VAS) [31]. The VAS consisted of a continuous line between two end-points, with 0 being "no pain" and 100 being "maximum tolerable pain".

### Range of motion assessment

A NedSGE/IBV electronic goniometer with one degree of freedom (Instituto de Biomecánica de Valencia, Valencia, Spain) was used to obtain the ROM. The assessment of active shoulder mobility of the injured arm was performed with the participants sitting on a rigid seat with a height-adjustable backrest, with their trunk upright, knees flexed at 90° and feet flat on the floor. The subjects’ pelvis, trunk and left arm were fastened with straps to fix their position, allowing movement of the right arm only and avoiding possible compensatory movements of the trunk [27]. The movements that were evaluated were flexion and abduction. Two repetitions of each movement were conducted and the average of these two measurements was used in further analyses. Goniometric measurements were performed by aligning the fulcrum of the goniometer with the corresponding location of the glenohumeral joint axis and aligning the goniometer arms with different bony landmarks depending on the movement. For flexion, the fulcrum used was the lateral aspect of the greater tubercle, the proximal arm of the goniometer was placed parallel to the midaxillary line of the thorax, and the distal arm was placed over the lateral midline of humerus (lateral epicondyle). For shoulder abduction, the fulcrum used was the anterior aspect of the acromion, the proximal arm was placed parallel to the midline of the anterior aspect of the sternum, and the distal arm was placed over the midline of the humerus [32]. The instructions were brief: “Lift your arm as much as you can and hold it in that position.” The static pose was held for five seconds.

### Isometric strength assessment

Two repetitions of flexion and abduction movements of the injured arm were assessed using a NedDFM/IBV portable dynamometer (Instituto de Biomecánica de Valencia, Valencia Spain). The individual was seated in the same way as for the ROM assessment. The dynamometer was placed just proximal to the elbow, with this joint flexed at 90°, and the resistance was offered by the physiotherapist [33], who was standing against a wall. The instructions were concise: “Push as hard as you can for five seconds” [34]. The mean force over this period of time was obtained. A one-minute rest was allowed between repetitions.

### Kinematic assessment

The kinematic assessment of scaption was performed using the Kinescan/IBV stereophotogrammetry system (Instituto de Biomecánica de Valencia, Valencia, Spain) with 4 CCTV cameras at 50 fps [27,35,36]. The individual was seated in the same way as for the previous assessments. The marker positions and trunk fastening are shown in Fig 1.

The trunk reference frame was defined in the initial posture using two markers on the left and right acromion processes (LA and RA, respectively), with the y-axis in the vertical direction, the x-axis perpendicular to the plane formed by the y-axis and the LA-RA line, and the z-axis pointing to the right. The motion of the trunk was tracked by a technical cluster of markers located on LA, the second dorsal vertebrae (D2) and the medial third of the scapular spine (SC). The right arm was tracked by a rigid three-marker cluster attached to the splint in the region of the forearm [27]. The starting position of the arm was fixed with the axis at 37.5° anteriorly to the coronal plane of the trunk, and 45° down the transverse plane of the shoulder, with the aid of a guide marked on a height-adjustable table and an electronic inclinometer.

The patients were asked to follow concise instructions: “At a comfortable speed, lift your arm as much as you can and hold it in the final position for three seconds. Then drop your arm back to the initial position”. Scaption was performed with two different loads, as follows: first 250 g and, after one minute rest, 1 kg. The data collection order was always the same for all subjects as [17,21],with the aim of avoiding fatigue that could be produced by carrying a heavy load. The maximum weight was established as 1 kg based on the capability shown by patients in previous studies [21]. Each load was lifted five times in a single trial.

### Data analysis

All kinematic data were exported, further processed and analyzed using custom routines in Matlab R2010a (MathWorks, Natick, MA, USA). The rotations of the thorax and the humerus from the starting position at any instant were calculated using Rodrigues’ vectors, following the procedures described in [27]. The XZ’Y” Euler sequence was chosen to represent the thoracohumeral motion, due to its better performance than YX’Y” in terms of reliability [27]. The resulting angles were smoothed and their time derivatives were calculated using the procedure described in [35]. Considering the lower reliability of the axial rotation and the plane of elevation angles for the motion being studied, only the first rotation *α*(*t*) (humeral elevation) and its angular velocity $\dot{\alpha }(t)$ were used in the subsequent analyses [27].

Only the ascent phase of the motion was analyzed. For each subject *i*, the velocity of the elevation angle ${\dot{\alpha }}_{i}(t)$ was used to split the j = 5 repetitions of the elevation gesture, obtaining *α*_*ij*_(*t*) and ${\dot{\alpha }}_{ij}(t)$. For each repetition j, the time base was normalized on a scale of 0 to 100 by means of a cubic spline representing the percentage of duration of each repetition, obtaining *α*_*ij*_(*n*) and ${\dot{\alpha }}_{ij}(n)$ [16].

The following dependent variables were calculated from the five repetitions of each movement for each subject: (i) average maximum humeral elevation (Emax), taking the average of the 5 repetitions Emax_j_ computed from *α*_*ij*_(*t*); (ii) variability of the maximum angle (VMA), as the coefficient of variation of the five peaks of maximum elevation Emax_j_; (iii) average maximum angular velocity (Velmax), taking the average of the 5 repetitions computed from ${\dot{\alpha }}_{ij}(t)$; (iv) average time to maximum velocity (tmaxvel), calculated taking into consideration the point in time (%) when the maximum velocity is achieved, computed from ${\dot{\alpha }}_{ij}(n)$ [37]; (v) repeatability, calculated as the reliability of the 5 repetitions of the normalized angular velocity functions ${\dot{\alpha }}_{ij}(n)$ by means of the ICC (intraclass correlation coefficient) [38].

### Statistics

All statistical analyses were performed with SPSS v.22 (IBM SPSS, Inc., Chicago, IL, USA). Standard statistical methods were used to obtain the mean and standard deviation (SD). Inferential analyses of the kinematic data were performed using two-way mixed multivariate analysis of variance (MANOVA), with an inter-subject factor called ‘injury’ with two categories (TG and RCTG) and a within-subject factor called ‘load’ with two categories (250 g and 1 kg). Post-hoc analysis was conducted using the Bonferroni correction provided by the statistics package used. Furthermore, to analyze the effect of the injury (i.e. TG and RCTG) on ROM and isometric strength, an independent Student’s t-test was conducted. We also compared the BMI and the level of pain experienced by both groups using the same statistical test. The type I error was established as < 5% (p < 0.05).

For each subject *i*, the repeatability was computed using ${\dot{\alpha }}_{ij}(n)$ waveform data as [38]:

$${ICC}_{i}\;({\dot{\propto }}_{i1}\;(n),\ldots ,{\dot{\propto }}_{iN}(n))=\frac{MST-MSE}{MST+(N-1)\times MSE}$$

Where *N* is the total number of j repetitions of shoulder elevation performed in the test (5 for this study), MST is the between-time mean square and MSE the within-time mean square from a one-way ANOVA per subject.

## Results

### Participants

The TG had a mean (SD) BMI of 27.52 (4.92) and a VAS score of 4.68 (2.56). The RCTG had a BMI of 28.24 (3.58) and a VAS score of 5.67 (1.22). There were no statistical differences between groups (p > 0.05). There were no participants with missing data.

### Range of motion

The results of the ROM comparison between the TG and RCTG, which are displayed in Table 1, showed that there were no significant differences in any of the movements assessed (i.e. flexion and abduction) (p > 0.05).

**Table 1:** Range of motion and isometric strength values in the tendinopathy and rotator cuff tear groups.

	TG	RCTG	P value
**ROM_flexion (°)**	118.37 (35.98)	104.89 (26.00)	0.81
**ROM_abduction (°)**	110.79 (38.60)	89.67 (15.56)	0.96
**IS_flexion (N)**	108.78 (52.82)	112.11 (43.03)	0.33
**IS_abduction (N)**	108.83 (60.93)	109.98 (60.17)	0.13

Data are shown as mean (standard deviation). TG = Tendinopathy group; RCTG = Rotator cuff tear group; ROM = range of motion; IS = isometric strength. P values are indicated for group comparisons for each variable.

### Isometric strength

The analyses of isometric strength demonstrate that there were no significant differences between the two groups (i.e. TG and RCTG) in abduction and flexion, as can be seen in Table 1 (p > 0.05).

### Kinematics

Table 2 displays the descriptive results and the post-hoc comparisons between levels of the two factors. As can be seen in the table, there were differences in the movement pattern performed with the two types of injury being studied, and they also show different behaviors with regard to the load lifted. In general, the Emax and Velmax values were lower in the RCTG than in the TG, during the movements performed with both 250 g and 1 kg. The variability (assessed with the VMA and repeatability) was greater in the RCTG than in the TG. Moreover, the average moment in the cycle at which the maximum velocity is achieved occurs earlier in the RCTG than in the TG, but only with 250 g. Regarding the differences in kinematic variables by load lifted, there were differences in Emax between the two loads for both injuries, with lower values for 1 kg than for 250 g. Furthermore, only the TG showed differences between loads in terms of Velmax and repeatability, both with lower values for 1 kg than for 250 g. On the other hand, the RCTG showed a significant increase in VMA and tmaxvel values with 1 kg than with 250 g.

**Table 2:** Differences in the kinematic variables between tendinopathy and rotator cuff tear groups and between the two loads (250 g and 1 kg).

	250 g		1 kg		p-values			
					Injury-based differences		Load-based differences	
	TG	RCTG	TG	RCTG	250 g	1 kg	TG	RCTG
Emax (°)	121.14 (6.51)	100.50 (9.46)	110.61 (7.72)	76.31 (11.22)	0.08	0.02	0.01	<0.01
VMA (%)	2.83 (.39)	3.61 (.56)	3.64 (.50)	5.51 (.73)	0.26	0.04	0.14	0.02
Velmax (°/s)	96.79 (12.94)	45.38 (18.80)	76.04 (11.60)	29.22 (16.85)	0.03	0.03	0.01	0.12
tmaxvel (%)	35.96 (1.85)	26.51 (2.69)	37.78 (2.14)	34.96 (3.11)	0.01	0.46	0.46	0.02
Repeatability (n.u.)	0.71 (.22)	0.54 (.18)	0.60 (.29)	0.45 (.26)	0.06	0.22	0.04	0.27

Data are shown as mean (standard deviation). Emax: maximum humeral elevation; VMA: variability of the maximum angle; Velmax: maximum angular velocity; tmaxvel: time to maximum velocity; n.u. = no units; TG: Tendinopathy group; RCTG: Rotator cuff tear group. P values are indicated for group and load comparisons.

**Fig 2:**
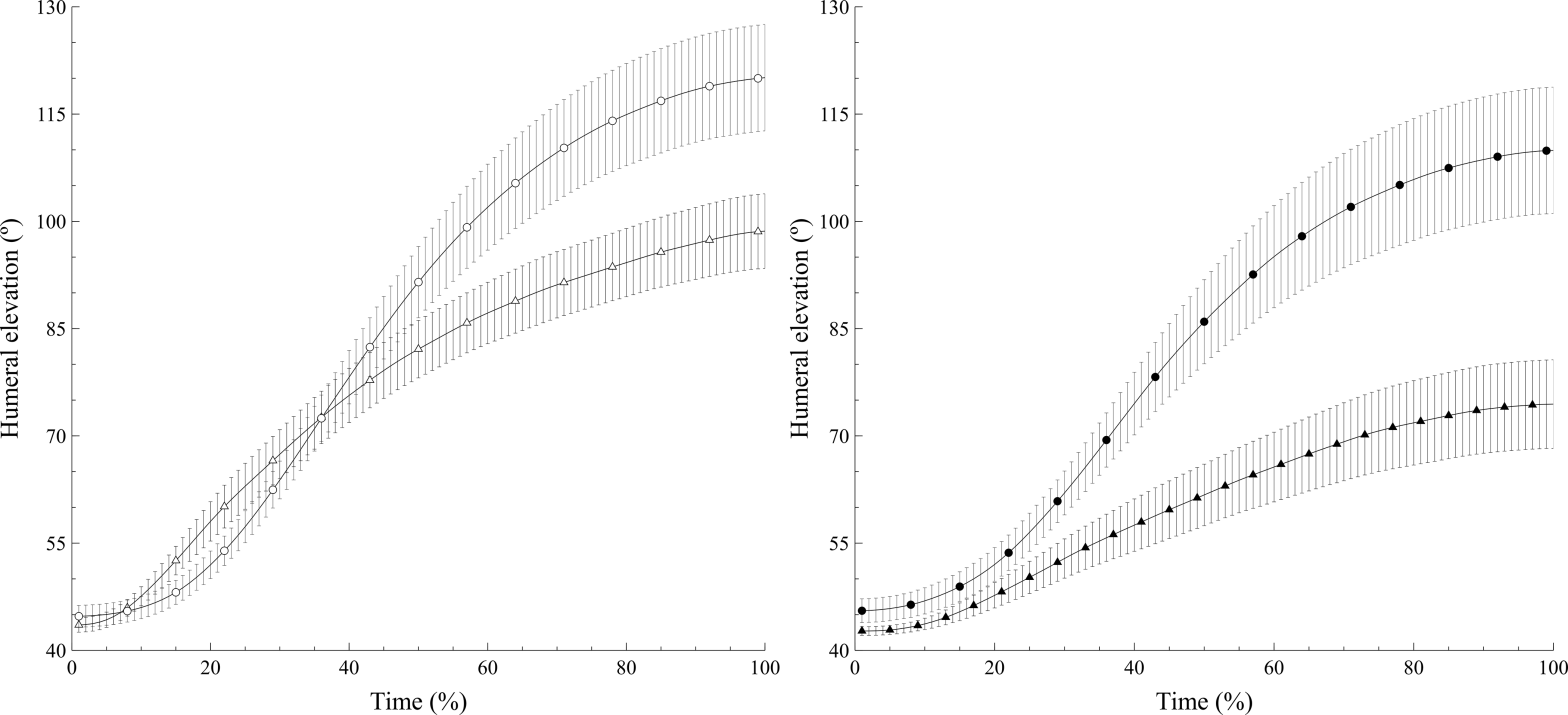
Mean humeral elevation curves in the tendinitis group (TG) and rotator cuff tear group (RCTG). The humeral elevation when lifting 250 g is shown in the left panel and when lifting 1 kg, in the right panel. The TG is represented by circles and the RCTG, by triangles. Filled shapes represent the 1 kg load and empty shapes, 250 g. Vertical lines represent the standard error of the mean.

Fig 2 shows the mean curves of the normalized humeral elevation angle (*α*(*n*)) for the TG and the RCTG. Although the two groups seem to present a different kinematic pattern in the elevation with 250 g, the differences in Emax did not reach the significance level (Table 2). A higher movement velocity of the RCTG could be deduced from the steeper slope of the curve, which should be related to the differences in tmaxvel. The plots also show the higher maximum elevation achieved by the TG with 1 kg and how the capacity to elevate the arm decreases as the handheld load increases in both groups (Table 2).

**Fig 3:**
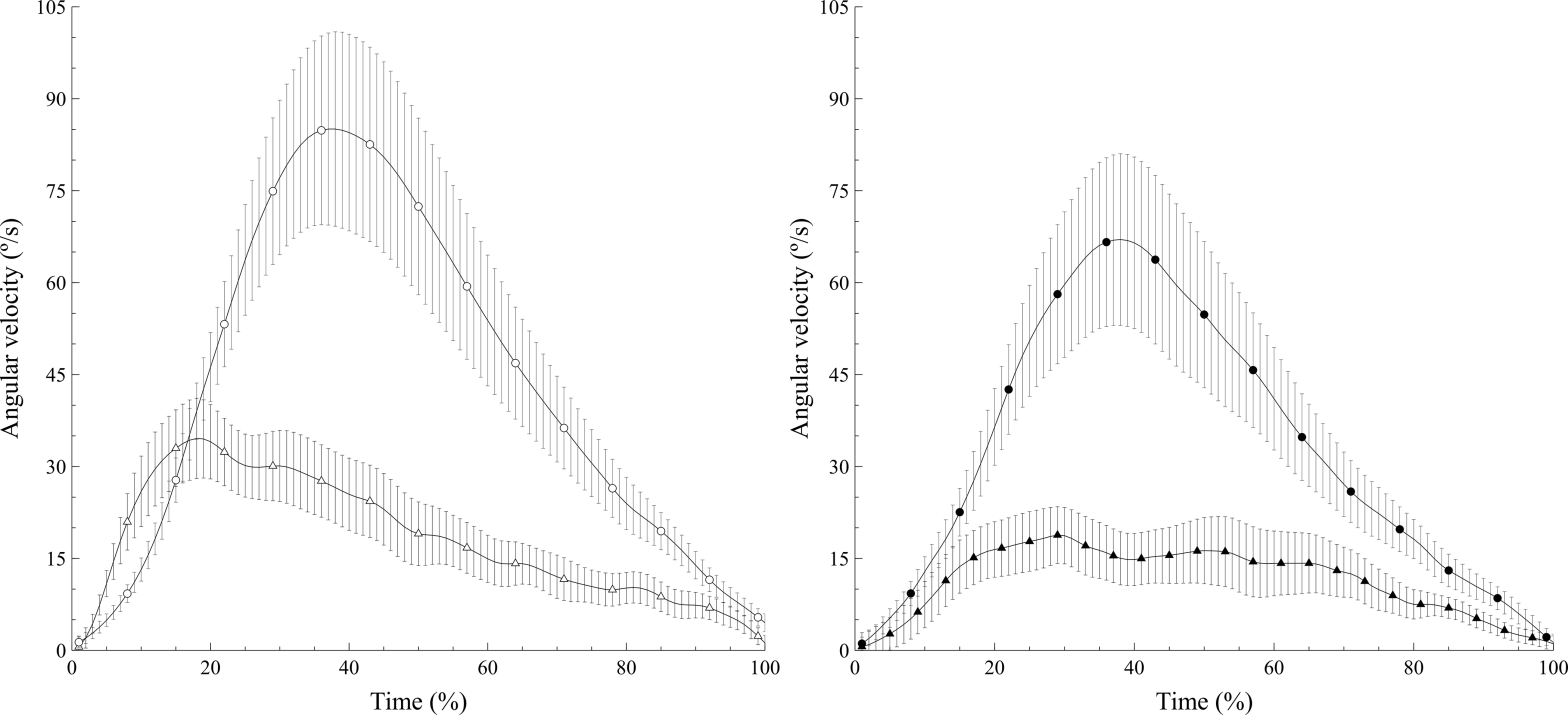
Mean curves of angular velocity in the tendinitis group (TG) and rotator cuff tear group (RCTG). The angular velocity when lifting 250 g is shown in the left panel and when lifting 1 kg, in the right panel. The TG is represented by circles and the RCTG by triangles. Filled shapes represent the 1 kg load and empty shapes, 250 g. Vertical lines represent the standard error of the mean.

Fig 3 shows the mean curves of angular velocity during humeral elevation for the TG and the RCTG. The figure graphically illustrates the different behavior observed in the two groups (Table 2). It can be observed that higher velocities were achieved during elevation in the TG compared with the RCTG, as can the influence of the load on Velmax values in the TG. It can also be seen that the time to maximum velocity occurs earlier in the RCTG than in the TG with 250 g (Table 2).

## Discussion

The kinematic variables measured in this study exhibit significantly different results during scaption depending on the shoulder injury in question and therefore provide information about the functional impact of the structural shoulder injury that is different from the information gained from the ROM variables obtained with goniometry and isometric strength assessed with a dynamometer. In our study, the Emax obtained is significantly lower in the participants with rotator cuff tear than in those with tendinopathy, although statistical significance is only reached when they lifted 1 kg, where the decrease is greater than 30%. Mell et al., who compared these two types of injuries using an electromagnetic tracking system, obtained a similar result, since people with rotator cuff tear presented lower values of humeral elevation than people with tendinopathy [16]. However, they did not find differences between their tendinitis group and a control group, probably because they studied a reaching motion that was limited to 100° of humeral elevation. Our TG performed a mean maximum elevation of 121.14°, which should be considered altered in comparison with the 163.3° achieved by a healthy group following exactly the same measuring procedures [27]. Furthermore, the effect of increasing the load placed in the hand during the scaption movement had a similar effect in both groups, meaning that both injuries are influenced by the load lifted, which makes it difficult to achieve the full ROM. Given that there are no previous in vivo studies analyzing the influence of load on thoracohumeral movement, these data cannot be directly compared with previous results. However, considering the scapula as part of the thoracohumeral movement, our results are consistent with previous studies that reported an influence of handheld weight on scapular kinematics [17,39].

With regard to Velmax, it can be seen that this variable presents lower values in the RCTG than in the TG with both 250 g and 1 kg. Scibek et al. observed that, in patients with rotator cuff tear, humeral elevation velocity increased when pain was reduced after a subacromial lidocaine injection [40]. However, the association between pain and velocity would not explain the lower Velmax in the TG, while there were no differences in the VAS score of the two groups. We suspect that people with a rotator cuff tear present greater functional impotence than people with tendinopathy. As a consequence, the most comfortable speed at which to perform shoulder scaption was lower in the RCTG than in the TG, regardless of the load lifted. When the Velmax is compared for the two loads, only the TG showed significant differences. Specifically, the Velmax decreases as the load increases. As can be seen in Table 1 and Fig 3, the Velmax is already very low in the RCTG when lifting 250 g, which explains why the difference is not statistically significant when comparing 250 g and 1 kg. It can therefore be seen that the ability of the RCTG to develop speed when moving light weights is compromised, regardless of the load.

Our results also show that the velocity pattern changes throughout the cycle of movement, which is represented by the tmaxvel. As can be seen in Fig 3, the average point in the elevation cycle at which the maximum velocity is reached occurs earlier in the RCTG (26.51%) than in the TG (35.96%) when lifting 250 g. Although their approach differs from ours, Mell et al. analyzed the mean slope of scapular elevation versus humeral elevation in scaption for three phases of the movement that they defined, as a measurement of velocity. They observed that the curve of scapulohumeral rhythm in patients with rotator cuff tear has a steeper slope in the early phases of elevation of the arm than in people with tendinopathy [16]. They justified this by attributing a greater contribution of the scapula than the glenohumeral movement in these early stages. Their findings may relate to the earlier tmaxvel observed in this group in our study. Hypothetically, peak velocity may be associated with an impulse with an upward translation of the scapula that initiates the movement (and leads to the glenohumeral movement) in order to develop the required angular momentum and reduce the pain associated with the glenohumeral movement. This hypothesis is based on the knowledge that tears in the supraspinatus lead to instability and pain [41] in the early stages of scaption because the supraspinatus muscle, together with the deltoid, are responsible for the early stages of the abduction movement [42]. However, the greater demand for strength when the load is increased to 1 kg probably does not allow this strategy to be adopted, which explains why statistical significance is not reached when the load is 1 kg. This may indicate that, when the load permits, patients perform this upward translation of the scapula as a compensatory strategy to conduct humeral elevation, and this strategy leads to a reduction in the contribution of the glenohumeral joint and/or reduces the pain experienced. Furthermore, the aforementioned authors associated the differences in scapulohumeral rhythm with a loss of strength and functionality in people with rotator cuff tear far more than with pain [16]. This conclusion concurs with our results, as there were no differences in perceived pain in our groups.

Movement variability (i.e. repeatability and VMA) was calculated in this study as a clinically interesting outcome due to its supposed association with the stability of the neuromuscular system [43]. Previous authors have shown altered movement variability in people with shoulder pain [44–46]. Although we did not find any differences in repeatability between the two pathological groups, we obtained lower values in the movement with 250 g in the TG (0.71) and the RCTG (0.54), compared with a healthy group (0.915) assessed with the same measuring procedures [27]. Furthermore, it is observed that repeatability decreases significantly when the load is increased to 1 kg in the TG, but not in the RCTG. These results may indicate that people with tendinopathy have a certain ability to adapt the movement to the associated physical demands. Thus, the TG may possess greater neuromuscular control when lifting 250 g than when lifting 1 kg. In contrast, the RCTG showed low repeatability of the movement, regardless of the load lifted, which indicates poor movement control even when the physical demand is low. Finally, our analysis showed that the VMA is increased in the RCTG when the movement is performed with 1 kg and is also higher than in the TG. This result reinforces the low motor control found in patients with rotator cuff tear, as they are not capable of achieving a reproducible maximum elevation with a 1 kg load, probably due to greater discomfort resulting from the injury.

We also analyzed the impact of these two shoulder injuries on ROM and isometric strength measured with a goniometer and dynamometer, respectively. Our results show that none of the variables obtained with these instruments show statistically significant differences between groups. This reinforces the previously explained idea that the static measurement of ROM and isometric strength is not comparable with dynamic information (i.e. velocity and quality of human motion), which could provide new insight in order to improve decisions regarding functional capacity, patient monitoring and prescription of rehabilitation strategies [10,40].

Our results are promising because the kinematic variables defined are capable of differentiating the functional impact of two similar shoulder injuries; therefore, it could be the first step in the development of shoulder kinematic patterns depending on the etiology. As limitations, it is important to point out that only the scaption movement, and only during the upward part of the cycle, was assessed in our study. Further, more information needs to be obtained about other types of injuries with a different functional impact (e.g. frozen shoulder, shoulder instability) and comparing the outcomes with a group of people without shoulder injury. In future studies, the analysis of more complex movements, for example daily living activities, should be taken into account as may be more appropriate to find functional differences between shoulder injuries. Furthermore, the results of our study should be considered with caution due to the sample size, because we may be committing a type II error. It would be necessary to conduct studies with a larger sample size in order to reach more specific conclusions about the clinical utility of kinematic assessment for shoulder injuries.

## Conclusions

Kinematic variables derived from the scaption motion assessment allow us to establish significant differences between individuals with tendinopathy and rotator cuff tear and therefore provide information about the functional impact of the shoulder injury. RCTG showed a lower active range of motion and velocity compared with the TG, as well as an alteration of thoracohumeral rhythm and an increase in variability. Further, these differences seem to be independent of the pain experienced as both groups presented a similar level of pain intensity. Moreover, the increase in the load lifted influences the movement pattern in both the TG, in which a decrease in Emax and velocity was experienced, and the RCTG, in which there was not only a decrease in Emax, but also an alteration in the velocity pattern and an increase in variability. Unlike dynamic variables, the static outcomes obtained with the goniometer and dynamometer did not show differences between the groups, which would indicate that dynamic information about the thoracohumeral motion is more appropriate to evaluate the functional status of the shoulder. Kinematic analysis may contribute to a better understanding of the impact of shoulder injuries, which would help in the assessment and treatment of shoulder pain.

## Supporting information

S1 File Database. (XLSX)
